# The UCMD-Causing *COL6A1* (*c*.930 + 189*C* > *T*) Intron Mutation Leads to the Secretion and Aggregation of Single Mutated Collagen VI *α*1 Chains

**DOI:** 10.1155/2023/6892763

**Published:** 2023-09-06

**Authors:** Carolin D. Freiburg, Herimela Solomon-Degefa, Patrick Freiburg, Matthias Mörgelin, Véronique Bolduc, Sebastian Schmitz, Pierpaolo Ala, Francesco Muntoni, Elmar Behrmann, Carsten G. Bönnemann, Alvise Schiavinato, Mats Paulsson, Raimund Wagener

**Affiliations:** ^1^Center for Biochemistry, Medical Faculty, University of Cologne, Cologne, Germany; ^2^Institute of Biochemistry, Faculty of Mathematics and Natural Sciences, University of Cologne, Cologne, Germany; ^3^Colzyx AB, Medicon Village, SE-223 81 Lund, Sweden; ^4^Neuromuscular and Neurogenetic Disorders of Childhood Section, Neurogenetics Branch, National Institute of Neurological Disorders and Stroke, NIH, Bethesda, Maryland, USA; ^5^Dubowitz Neuromuscular Centre, UCL Great Ormond Street Institute of Child Health & Great Ormond Street Hospital NIHR Biomedical Research Centre, London, UK; ^6^Center for Molecular Medicine (CMMC), Cologne, Germany; ^7^Cologne Center for Musculoskeletal Biomechanics (CCMB), Cologne, Germany

## Abstract

Collagen VI is a unique member of the collagen family. Its assembly is a complex multistep process and the vulnerability of the process is manifested in muscular diseases. Mutations in *COL6A1*, *COL6A2*, and *COL6A3* lead to the severe Ullrich Congenital Muscular Dystrophy (UCMD) and a spectrum of disease of varying severity including the milder Bethlem muscular dystrophy. The recently identified dominant intronic mutation in *COL6A1* (*c*.930 + 189*C* > *T*) leads to the partial in-frame insertion of a pseudoexon between exon 11 and exon 12. The pseudoexon is translated into 24 amino acid residues in the N-terminal region of the triple helix and results in the interruption of the typical G-X-Y motif. This recurrent *de novo* mutation leads to UCMD with a severe progression within the first decade of life. Here, we demonstrate that a mutation-specific antibody detects the mutant chain colocalizing with wild type collagen VI in the endomysium in patient muscle. Surprisingly, in the cell culture of patient dermal fibroblasts, the mutant chain is secreted as a single *α* chain, while in parallel, normal collagen VI tetramers are assembled with the wild-type *α*1 chain. The mutant chain cannot be incorporated into collagen VI tetramers but forms large aggregates in the extracellular matrix that may retain the ability to interact with collagen VI and potentially with other molecules. Also, *α*1 chain-deficient WI-26 VA4 cells transfected with the mutant *α*1 chain do not assemble collagen VI tetramers but, instead, form aggregates. Interestingly, both the wild type and the mutant single *α*1 chains form amorphous aggregates when expressed in HEK293 cells in the absence of *α*2 and *α*3 chains. The detection of aggregated, assembly incompetent, mutant collagen VI *α*1 chains provides novel insights into the disease pathophysiology of UCMD patients with the *COL6A1* (*c*.930 + 189*C* > *T*) mutation.

## 1. Introduction

The ubiquitously expressed microfibrillar collagen VI anchors large interstitial structures and cells [1]. The collagen VI *α* chains are encoded by six genes, and the predominant form contains the *α*1, *α*2, and *α*3 chains. The remaining *α*4, *α*5, and *α*6 chains have very specific expression patterns in mouse [2] and can replace the *α*3 chain in the heterotrimers [3, 4]. In humans and modern apes, the *α*4 chain encoding gene is inactivated due to a pericentric inversion on chromosome 3 resulting in two pseudogenes [4, 5].

The *α* chains of collagen VI share, compared to fibrillar collagens, an exceptionally short collagenous region of 336 amino acid residues carrying repeated imperfections in the Gly-X-Y motif. The N- and C-terminal globular regions mainly consist of VWA domains, which are prominent cell adhesion and protein-protein interaction domains [6]. All *α* chains contain two C-terminal VWA domains, and the *α*5 chain an additional VWA domain. At the N-terminus the *α*1 and *α*2 chains carry a single and the *α*3- *α*6 chains seven to ten VWA domains. Therefore, collagen VI is rather a VWA multimer than a classical collagen. The larger *α*3 chain has additional domains at the very C-terminus: a unique region, a fibronectin III repeat, and a Kunitz domain [7, 8]. This C-terminal region of the *α*3 chain is cleaved by BMP-1, furin, and MMP14 [9–11]. The cleaved-off Kunitz domain was named endotrophin and proposed to act as a matrikine involved in cancer progression, fibrosis, and insulin resistance [12, 13].

Collagen VI is assembled in a multistep process and is, unlike other collagens, assembled into higher oligomers prior to secretion. The three different *α* chains form triple helical monomers. Dimers form in an antiparallel manner driven by an interaction between the C2 domain of the *α*2 chain and the triple helical region [14, 15]. The dimers are staggered with an overlapping segmented supercoil of 75 nm, leaving 15 nm of nonoverlapping triple helix at each end [16]. Two dimers assemble laterally by nucleating at the N-terminal end and form collagen VI tetramers of about 2000 kDa. These are further stabilized by disulphide bonds between cysteines in the nonoverlapping triple helical part of the *α*3 chain [15]. Tetramers are secreted and associated end-to-end by noncovalent interactions of the flanking globular domains into linear microfibrils with a periodicity of 112 nm [17, 18]. MIDAS motifs in the C-terminal VWA domains of the *α*1 and *α*2 chains play an essential role in the correct formation of tetramers and microfibrils, probably by facilitating intramolecular interactions [14, 19]. The N5 and C5 domains of the *α*3 chain are crucial for efficient microfibril formation, but the C2-C5 domains are cleaved off after microfibrils have been formed [20, 21]. Modified, linear microfibrils are laterally connected into a collagen VI network by interacting with other extracellular matrix proteins [16, 22, 23]. The structural arrangement of the N-terminal domains of the long collagen VI chains (*α*3-*α*6) implies a flexible character and a functional adaptability of these domain arrays [24].

Mutations in *COL6A1*, *COL6A2*, and *COL6A3* lead to a spectrum of collagen VI-related muscular dystrophies, ranging from the severe Ullrich congenital muscular dystrophy (UCMD) via intermediate phenotypes to the milder Bethlem muscular dystrophy (BM). UCMD has an early onset and is typically detected already at birth. Frequent features are muscle weakness, hypotonia, scoliosis, proximal joint contractures, and distal joint laxity. Most patients are never able to walk or lose ambulation as teenagers due to progressive muscle weakness and joint contractures. Thanks to nocturnal respiratory support, patients routinely reach adulthood [25–27]. BM with milder symptoms is often recognized first in adulthood, although it can be traced back to early childhood. Typical symptoms are contractures in long finger flexors and in the wrist, elbow, and Achilles tendons. Contractures and muscle weakness lead to limited mobility and patients over 50 years of age often need walking aids [26]. BM and UCMD share variable skin phenotypes, e.g., keratosis pilaris, abnormal scarring (keloid or atrophic scars), and abnormal keratinisation that results in rough skin texture [28]. In some cases, due to overlapping symptoms, neither molecular diagnosis nor clinical presentation allows the clear distinction between the two myopathies. These patients have intermediate phenotypes and are diagnosed with mild UCMD or severe BM [29]. BM was first described as an autosomal dominant inherited disorder [30] while UCMD as a recessive condition and many attempts were made to categorize the myopathies by the mode of inheritance. Still, the two forms cannot be separated on the mode of inheritance only, as either dominant or recessive mutations can cause both BM and UCMD [15, 26].

Muscle biopsies are often used for the histological characterization of UCMD and BM patients. The findings vary from almost normal to myopathic muscle structures with variable fiber diameter, atrophic fibers, and an increase of surrounding connective and adipose tissue [26]. In the case of recessive mutations in UCMD, staining by collagen VI antibodies is lost or severely reduced, whereas, with dominant mutations, the extracellular matrix staining for collagen VI no longer colocalizes with basement membrane markers. These findings imply that not only the lack of collagen VI but also its missing interactions with other extracellular matrix proteins can result in severe phenotypes.

Most commonly, collagen VI myopathies are due to dominant gain of misfunction mutations in the triple helical region, mostly glycine substitutions or splicing mutations in its N-terminal part. Loss-of-function mutations in COL6A1 are those that either introduce premature stop codons—nonsense mutations and frameshifts—or that affect glycine residues in the C-terminal part of the triple helix and thereby prevent its assembly. Homozygous premature stop codon mutations in *COL6A1*, *COL6A2*, and *COL6A3*; thus the complete loss of collagen VI leads to severe phenotypes [31–33].

Here, we explored the consequences of a severe UCMD-causing *COL6A1* (*c*.930 + 189*C* > *T*) dominant deep intronic splice activating mutation [34] on the assembly of collagen VI. The mutation creates a novel consensus splice site donor site, as is most frequently observed in pseudoexon pathogenesis [35]. This causes the inclusion of a pseudoexon in the final mRNA transcript that codes for an insertion of 24 amino acid residues in the N-terminal region of the triple helix and results in the interruption of the typical G-X-Y motif. Pseudoexon activation is a heavily underreported disease mechanism that cannot only be caused by the creation of *de novo* pseudo splice sites but also by strengthening preexisting pseudo splice sites, by alterations to the splicing regulatory environment, by genomic rearrangements, or by loss of splice site competition due to inactivation of authentic splice sites [36]. Our results provide new insights into the molecular basis of the disease-causing expression of the pseudoexon-containing mRNA transcript.

## 2. Experimental Procedures

### 2.1. Affinity Purification of the Pex11-*α*1(VI) Antiserum

The generation of the Pex11-*α*1 (VI) antiserum was earlier described [37]. The antiserum was affinity purified by coupling the pseudoexon peptide (TRSTAPRRPLHLEGQGQPPRHPAK-C) to a SulfoLink column (Thermo Scientific). Coupling and subsequent purification were performed according to the producer's manual. Briefly, 100 *μ*g peptide in coupling buffer containing 25 mM TCEP was coupled to the SulfoLink resin and equilibrated with coupling buffer. The antiserum was diluted in coupling buffer, centrifuged, and the supernatant passed over the SulfoLink column. After washing with PBS, the purified antibody was eluted from the column with 0.2 M glycine, pH 2.5, and neutralized by adding 50 *μ*l 1 M Tris, pH 8.8.

### 2.2. cDNA Cloning, Transfection, and Recombinant Protein Expression

The wild-type full-length collagen VI *α*1 chain sequence was amplified by PCR on human cDNA with the following primers: 5′-CGCGCTAGCGACTGCCCCGTGGACCT-3′ (forward), 5′-CTAGGATCCTTAGCCCAGCGCCACCTT-3′ (reverse). By cloning using *BstBI* (5′) and *BsiWI* (3′) restriction sites, a 1300 bp synthetic DNA fragment (Supporting Information: Table S1) containing the mutant sequence (GeneArt String, Invitrogen) replaced the respective wild-type sequence and was amplified by PCR. Wild type and mutant PCR products were cloned into a modified pCEP-pu vector containing an N-terminal BM40 signal peptide and an N-terminal One-STrEP-tag or an N-terminal His_8_ tag [38, 39]. The recombinant plasmids were introduced into HEK293-EBNA cells (Invitrogen) using the FuGENE 6 transfection reagent (Roche Applied Science). Cells were selected with puromycin (1 *μ*g/ml), and the recombinant proteins purified directly from serum-free culture medium. For the generation of stable WI-26 VA4 cell lines, the PCR products were cloned into a modified sleeping beauty vector [40, 41]. Recombinant proteins carrying the His_8_ tag were used for immunofluorescent microscopy. Recombinant proteins carrying a One-STrEP-tag were purified from cell culture supernatants with a Strep-Tactin® XT column (IBA). Expression and purification of recombinant collagen VI tetramers are described elsewhere (Schiavinato et al., in preparation).

### 2.3. Collagen VI *α*1 Chain Antibody Generation

The purified recombinant Strep-tagged wild-type human collagen VI *α*1 chain was used to immunize rabbits and guinea pigs (Pineda Antibody Service). The antisera obtained were affinity purified on a column with the antigen coupled to CNBr-activated Sepharose 4B column (GE Healthcare). The bound antibodies were eluted with 0.1 m glycine, pH 2.5, and the eluate was neutralized with 1 m Tris-HCl, pH 8.8, and adjusted to 150 mM NaCl.

### 2.4. Cell Culture

Control dermal fibroblasts and such from patients that carry the *COL6A1* (*c*.930 + 189*C* > *T*) mutation (R1, US8, US10; NIH) [37], HEK293-EBNA and WI-26 VA4 cells were cultured in Dulbecco's modified Eagle's medium (DMEM) or Ham's F-12 nutritional mix. Media were supplemented with 10% FCS and 1% penicillin/streptomycin. Stable cell lines were generated by transfection with FuGENE® HD Transfection Reagent (Promega) and selection with 2 *μ*g/ml puromycin. Ascorbic acid (0.55 mg/ml ascorbic acid, 0.1625 mg/ml ascorbic acid-2- phosphate) was added to the cells every second day.

### 2.5. Circular Dichroism Spectroscopy

The samples (100 *μ*g/ml) were dialysed overnight against a 10 mM phosphate buffer, pH 7.4. A JASCO-715 spectrometer was used along with a 0.1 cm cuvette at 20°C. In order to exclude background signals, the buffer was measured first as a blank control. Subsequently, the protein solutions were measured in the same cuvette at a speed of 5 nm/min and a wavelength step of 0.5 nm. Five spectra were recorded and averaged. The data was analyzed using the DichroWeb online server (http://dichroweb.cryst.bbk.ac.uk/html/home.shtml) providing calculated secondary structure content.

### 2.6. Electron Microscopy

Immunogold electron microscopy was performed as previously described [10]. In brief, samples of dermal fibroblast (US8; NIH) cell culture supernatant were adsorbed onto a polymer surface and incubated with the 5 nm colloidal gold labelled Pex11-*α*1(VI) antibodies and 10 nm colloidal gold labelled antibodies against the N-terminal region of the human collagen VI *α*3 chain (h*α*3N) [42] for 30 min at 37°C with subsequent negative staining.

For single particle negative stain EM, affinity purified proteins were applied to freshly glow-discharged carbon grids (R2/1 + 2 nm C, Cu 200^∗^, Quantifoil*).* Excess protein was blotted away with Whatman paper, the grids were washed with H_2_O, and the remaining protein was stained with uranyl formate (0.75% w/v). Excess liquid was removed by blotting, and the remaining thin layer of stain was rapidly dried by a laminar flow of air. Transmission electron microscopic images were acquired on a Talos L120C (ThermoFischerScientific) microscope operating at 120 kV, equipped with a Ceta camera. Alignment and astigmatism correction were performed manually using the microscope's user interface, and then images were acquired using low-dose condition settings in EPU.

### 2.7. Immunofluorescence Microscopy

Immunofluorescence microscopy was performed on frozen sections of muscle biopsies, primary fibroblasts, or WI-26 VA4 cells. Sections (NH-441) were supplied by the MRC Centre for Neuromuscular Diseases Biobank London (UCL, London, UK; Research Ethics Committee reference no. 06/Q0406/33). The presence of the *COL6A1* (*c*.930 + 189*C* > *T*) mutation was confirmed by DNA sequencing (data not shown). For all samples collected by the Biobank after 01/09/2006, written consent for research has been obtained from patients or their parent/guardian. All samples were supplied to the project anonymized. Frozen sections were thawed, washed twice with TBS, and fixed with methanol/acetic acid (95%/5%) solution. Cells were fixed with 4% PFA in PBS and washed with PBS. Muscle sections were blocked with 5% BSA/TBS and cell cultures with 1% FCS/PBS. If permeabilization was desired, sections and cells were incubated with 0.1% NP-40/TBS or PBS. Primary antibodies were diluted in blocking solution and incubated for 1 h at room temperature. After washing, fluorophore-conjugated secondary antibodies (Alexa Fluor 555-goat anti-guinea pig, Alexa Fluor 488-goat anti-rabbit) and DAPI were added to sections or cells and incubated for 45 min. After repeated washing, the sections were covered with DAKO mounting medium (Agilent) and coverslips. Cells grown on coverslips were mounted with DAKO mounting medium (Agilent) and placed inverted on microscope slides. Images were taken with the TCS SP5 confocal laser scanning microscope (Leica). The following primary antibodies were used: guinea pig anti-ColVI*α*3N [42], rabbit anti Pex-11-*α*1(VI) (in house), guinea pig anti-ColVI-*α*1 (in house), rabbit anti-ColVI*α*3 C5 [10], and mouse anti-His_4_ (Qiagen).

### 2.8. Size Exclusion Chromatography of Recombinant Wild Type and Mutant Collagen VI *α*1 Chains

A Superose 6 Increase 10/300 GL column in an ÄKTA FPLC system was equilibrated in filtered, degassed 1x TBS with a flow rate of 0.5 ml/min. In order to remove aggregated protein, the solution was applied to a Spin-X centrifugation tube with a pore size of 0.22 *μ*m (Corning) and centrifuged. 300 *μ*l of the sample was loaded via a 200 *μ*l loop onto the column with a flow rate of 0.3 ml/min. The absorbance was measured at 280 nm.

### 2.9. SDS-PAGE, Coomassie Staining, and Immunoblotting

Cell culture lysates and supernatants were supplemented with SDS-sample buffer with or without 5% *β*-mercaptoethanol and subjected to SDS-PAGE or composite 0.5% agarose/2.4% polyacrylamide gel electrophoresis. Proteins separated by SDS-PAGE were stained overnight with Coomassie Brilliant Blue G250 and destained. For immunoblotting, the proteins were transferred onto a nitrocellulose membrane (0.2 *μ*m; GE Healthcare) and blocked with 5% milk powder in TBS. Membranes were first incubated with primary antibodies and then with horseradish peroxidase-conjugated secondary antibodies in a blocking buffer (rabbit anti-guinea pig and rabbit anti-mouse; Invitrogen). Signals were detected by chemiluminescence (ECL, Cytiva) in the ImageQuant 800 Fluor imager (Cytiva). The following primary antibodies were used: guinea pig anti-ColVI*α*3N [42], rabbit anti-Pex-11-*α*1(VI) (in house), guinea pig anti-ColVI-*α*1 (in house), and rabbit anti-ColVI*α*3C5 [10].

### 2.10. Generation of WI-26 VA4 *COL6A1* CRISPR-Cas Knockout Cells

The CRISPR/Cas9 system was used to ablate *COL6A1* in Wl-26 VA4 cells [43]. A 20 nt guide sequence was designed using the CRISPOR tool (http://crispor.tefor.net/). To generate the oligonucleotide duplex, 3 *μ*L 5′-CACCGTTGTCCACGAGGGCCCCGTA-3′ (sense); 5′-AAACTACGGGGCCCTCGTGGACAAC-3′ (antisense) oligonucleotides (100 *μ*M) targeting exon 2 of *COL6A1* were mixed with 39 *μ*L H_2_O and incubated for 5 min in boiling water. After cooling the reaction for 15 min at RT, 5 *μ*L 10x T4 ligation buffer (Thermo Scientific) and 1 *μ*L T4 polynucleotide kinase PNK (NEB) were added and incubated for 30 min at 37°C. 1 *μ*g of the vector pSpCas9(BB)-2A-Puro V2.0 was linearized with the restriction enzyme Bbsl and purified with the DNA NucleoSpin Gel and PCR Clean-up kit (Macherey-Nagel). The oligonucleotide duplex and vector were ligated and directly transformed into *E.coli*, and the vector was purified with standard methods. Wl-26 VA4 cells were transfected with the Cas9/gRNA vector using the FuGENE 6 transfection reagent (Roche). Preselected cells were cultured with 2 *μ*g/ml puromycin on a 10 cm dish, detached with trypsin/EDTA, and resuspended in a 10 ml medium for further single-cell selection. Subsequently, the cell suspension was diluted 1 : 1000 and seeded on 10 cm dishes. The low cell density allowed the monitoring of single cells which grew within 1-2 weeks into single-cell colonies. These were isolated with PYREX® cloning cylinders (Corning), detached with trypsin/EDTA, and incubated for 10 min at RT. 15-30 colonies were transferred into 12-well plates and cultured until reaching confluency. Single cells were screened by PCR on gDNA (AGGCCCAGCAGAGACTCG forward; CCTTAGGAGGTTGAGGCCGT reverse) and immunoblot.

## 3. Results

### 3.1. The Affinity Purification of the Pex11-*α*1(VI) Antibody Generates a Highly Specific Tool to Study the Pathomechanism of the UCMD-Causing *COL6A1* (*c*.930 + 189*C* > *T*) Intron Mutation

**Figure 1:**
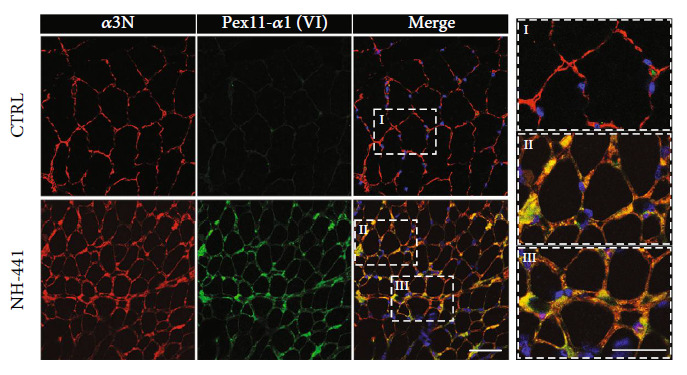
Immunofluorescence microscopy of muscle sections from control (CTRL) and *COL6A1* (*c*.930 + 189*C* > *T*) patients. Stainings (patient NH-441) were performed with an antibody against the N-terminal part of the *α*3 chain (ColVI*α*3N, in red) and the affinity purified Pex11-*α*1(VI) antibody (green). Nuclei were stained with DAPI (blue). Fields marked by white frames are further magnified. Scale bar = 50 *μ*m. The images show the mutant *α*1 chain is localized to endomysium and perimysium, often in strongly fibrotic areas.

**Figure 2:**
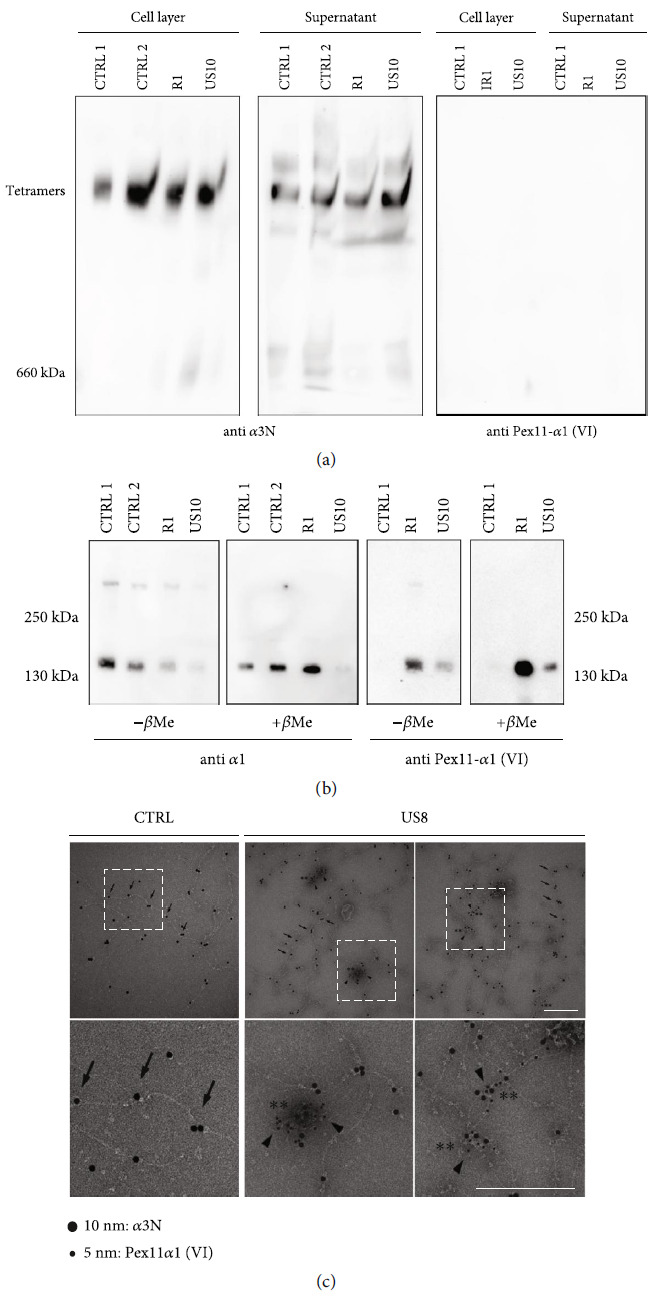
Secretion of collagen VI tetramers and non-assembled mutant *α*1 chains by patient dermal fibroblasts. (a) Duplicates of samples from the cell layer and supernatants of control (CTRL) and patient fibroblasts (R1, US10) were subjected to electrophoresis in composite 0.5% agarose/2.4% polyacrylamide gels. Proteins were transferred to a membrane and signals were detected with the *α*3N and the Pex11-*α*1 antibodies. (b) Supernatants from (a) were subjected to SDS-PAGE on 6% gels under nonreducing (-*β*Me) or reducing (+*β*Me) conditions. (c) Negative stain electron micrographs of immunogold labelled supernatants of control and patient fibroblasts (US8). The *α*3N antibody was labelled with 10 nm and the Pex11-*α*1 antibody with 5 nm gold particles. Fields marked by white frames are further magnified in the bottom row. Arrows mark the periodic beaded region of collagen VI visualized by *α*3N signals, arrowheads point to Pex11-*α*1 signals, and tangles of mixed *α*3N and Pex11-*α*1 signals are indicated by asterisks. Scale bar = 200 nm. The results indicate that, despite being present, the mutant chains cannot assemble into tetramers but form aggregates and, sometimes, decorate collagen VI microfibrils.

It was earlier shown that a heterozygous mutant collagen VI *α*1 chain carrying the pseudoexon encoded 24 amino acid residue insertion in the triple helical region can be secreted [37]. To study the consequences of the mutation, we earlier generated the polyclonal antiserum [Pex11-*α*1(VI)] that detects the translated pseudoexon [37]. For the present study, the antiserum was affinity purified by using the immunizing peptide coupled to a SulfoLink column (Thermo Scientific). The specificity of the affinity purified antibodies was unequivocally demonstrated by immunohistochemistry and immunoblotting on patient material (Figures 1 and 2).

### 3.2. The Mutant Collagen VI *α*1 Chains Colocalize with Wild-Type Collagen VI in the Endomysium of *COL6A1* (*c*.930 + 189*C* > *T*) Patient Muscle

To better understand the consequences of the heterozygous *COL6A1* (*c*.930 + 189*C* > *T*) intronic mutation, the localization of the mutant chain in its physiological setting was studied by immunofluorescence microscopy on sectioned patient muscle biopsies. An antibody against the collagen VI *α*3 chain N-terminal region (*α*3N) stained a severely perturbed collagen VI matrix in the affected muscle. The patient's muscle also showed typical dystrophic muscle fibers of enlarged and irregular diameter as well as an enlarged endomysium and perimysium. The Pex11-*α*1(VI) antibody, specific for the mutant chain, gave signals only in muscle sections from the *COL6A1* (*c*.930 + 189*C* > *T*) patients. Costaining with the *α*3N and the Pex11-*α*1(VI) antibodies showed a broad overlap of signals, indicating an extracellular colocalization of the mutant *α*1 chain and wild-type collagen VI (Figure 1) in patient muscle tissue. The mutant *α*1 chain was mainly localized to the endomysium and perimysium surrounding muscle fibers and appeared to coincide with strongly fibrotic areas (Figure 1).

### 3.3. Collagen VI Tetramers and Nonassembled Mutant *α*1 Chains Are Secreted by *COL6A1* (*c*.930 + 189*C* > *T*) Patient Dermal Fibroblasts

However, immunofluorescence microscopy did not answer the question of how wild-type collagen VI and the mutant chain assemble. As access to patient muscle specimens is limited and as collagen VI is expressed by the muscle interstitial fibroblasts, patient dermal fibroblasts were isolated and cultured, and cell culture supernatants and cell layer extracts were subjected to composite agarose/polyacrylamide gel electrophoresis and immunoblotting. The *α*3N antibody detected collagen VI tetramers in the cell lysate and in the cell culture supernatant, showing that the mutant chains do not prevent collagen VI tetramer formation. When the Pex11-*α*1(VI) antibody was applied, samples of cell lysate and supernatant did not show a signal at the height of tetramers, indicating that the mutant chains cannot assemble into larger structures (Figure 2(a)). In normal SDS-PAGE under both reducing and nonreducing conditions, the free mutant *α*1 chains were detected in cell culture supernatants (Figure 2(b)).

### 3.4. Secreted Mutant Collagen VI *α*1 Chains Form Unordered Aggregates Which Can Also Associate with Collagen VI Microfibrils

To determine the influence of the mutant *α*1 chains on the assembly of collagen VI tetramers into microfibrils and to unravel the organization of the secreted mutant chains, immunoelectron microscopy after negative staining was applied to supernatants from control and patient dermal fibroblast cultures. The Pex11-*α*1(VI) antibody was labelled with 5 nm and the *α*3N antibody with 10 nm colloidal gold particles (Figure 2(c)). The electron micrographs showed wild-type collagen VI microfibrils with the typical spacing of the *α*3 chain N-termini detected by the *α*3N antibody in both control and patient samples. In the patient samples, the Pex11-*α*1(VI) 5 nm gold particles were sometimes seen in protein aggregates that were not labelled by the *α*3N antibody. However, the Pex11-*α*1(VI) antibody also decorated collagen VI microfibrils (Figure 2(c)).

### 3.5. The Mutant Collagen VI *α*1 Chains Colocalize with the Extracellular Collagen VI Network in Cultures of Patient Dermal Fibroblasts

**Figure 3:**
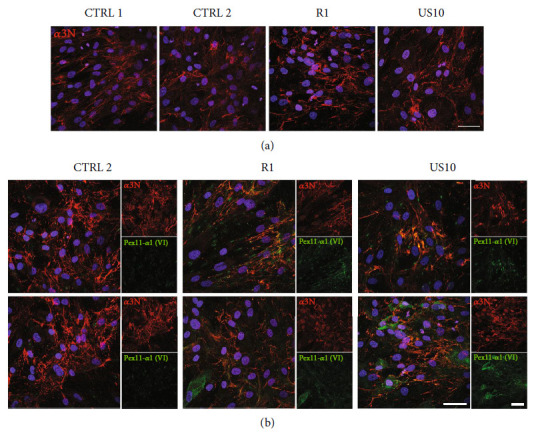
The mutant chains associated with the collagen VI network in fibroblasts from *COL6A1* (*c*.930 + 189*C* > *T*) patients. Patient fibroblasts (R1, US10) and control fibroblasts were incubated with ascorbic acid for five days. The *α*3N antibody was used to visualize the collagen VI network (a, b) and the affinity purified Pex11-*α*1(VI) antibody to show mutant chains (b). Nuclei were stained with DAPI (blue), scale bar = 50 *μ*m. The mutant chains colocalize with the wild-type collagen VI network, showing their deposition in the extracellular matrix.

Patient fibroblasts were cultured on coverslips (Figures 3(a) and 3(b)) and labelled with the Pex11-*α*1(VI) antibody to study the extracellular localization of the mutant *α*1 chains by immunofluorescence microscopy (Figure 3(b)). The *α*3N antibody was used to show if there were differences in distribution compared to wild-type collagen VI (Figures 3(a) and 3(b)). In general, the formation of the collagen VI network was not affected. However, structural aberrations of the collagen VI microfibrils, which appeared discontinuous and kinked, have been described [37] and were visible also here. The Pex11-*α*1(VI) antibody signal sometimes merged with that of the collagen VI extracellular network, showing the deposition of the mutant chains in the extracellular matrix (Figure 3(b)).

### 3.6. Recombinantly Expressed Mutant *α*1 Chains Form Amorphous Aggregates

**Figure 4:**
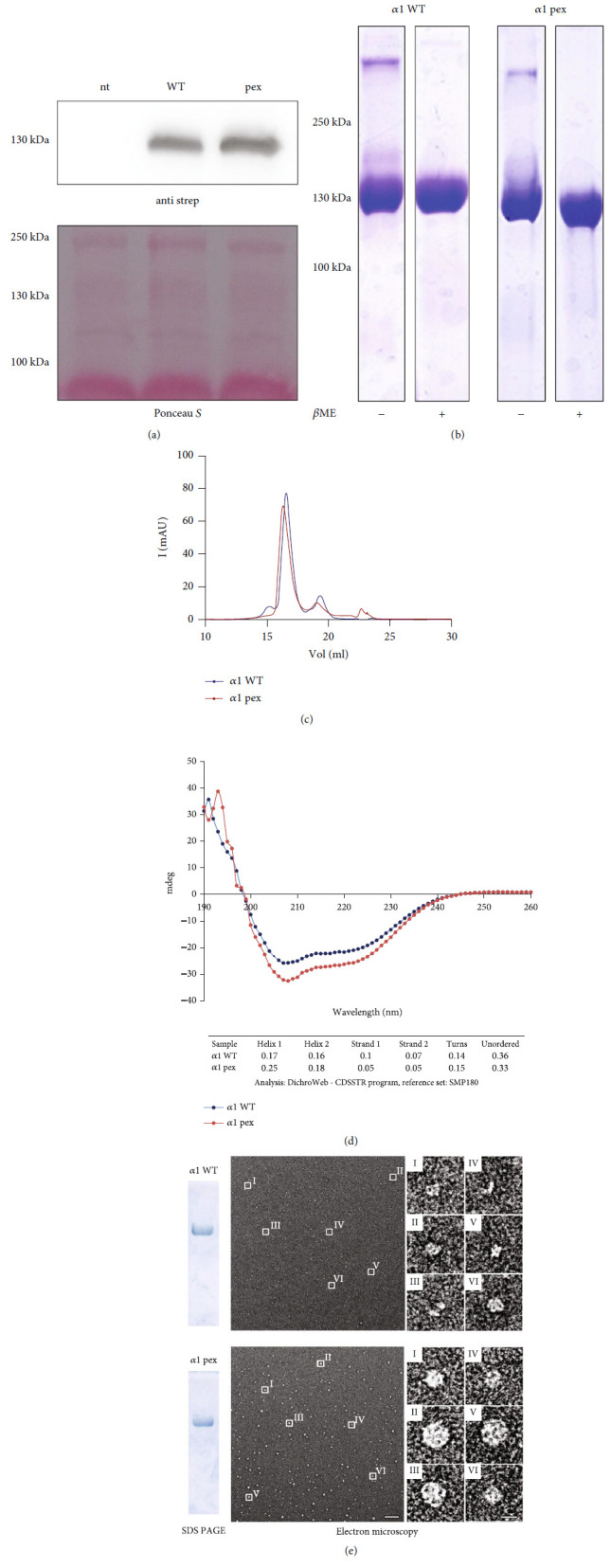
Purification and characterization of recombinantly expressed full-length *α*1 wild type and mutant chains. (a) Immunoblot with Ponceau S staining of supernatants from not transfected (nt) HEK293 EBNA cells, cells transfected with the wild-type chain (WT) or the mutant chain (Pex) detected with an anti-Strep antibody. (b) Coomassie-stained SDS-PAGE under nonreducing (-*β*Me) and reducing conditions (+*β*Me) after affinity purification. (c) Size exclusion chromatography of affinity purified samples. (d) Secondary structure analysis by circular dichroism spectroscopy and (e) Coomassie-stained SDS-PAGE gel of purified proteins and negative staining electron microscopy of single particles. Particles marked by white frames are further magnified. Scale bars = 100 nm in overviews and 10 nm in magnified images. The results indicate that the mutant chains are expressed and secreted and do not show gross structural aberrations. Under these conditions, both wild type and mutant chains tend to aggregate, but the aggregates formed by the mutant chains are larger.

To better understand the dominant-negative effect of the mutant collagen VI *α*1 chains, wild type and mutant *α*1 chains were recombinantly expressed in HEK293-EBNA cells and further characterized. HEK293-EBNA cells were chosen, as they do not express endogenous collagen VI. Their use, therefore, avoids problems due to an unbalanced expression of the different *α* chains. Wild type and mutant *α*1 chains were secreted in similar amounts (Figure 4(a)), indicating that expression and secretion of unassembled single full-length *α*1 chains occur with or without the mutation. The One-STrEP tagged proteins could be purified in high yield by affinity chromatography of cell culture supernatants (Figure 4(b)). Gel filtration did not show an obvious difference in the elution profile between the recombinant wild type and mutant *α*1 chains (Figure 4(c)). Circular dichroism spectroscopy was performed to determine the secondary structure (Figure 4(d)) but did not detect significant differences between the wild type and mutant chains. The spectra indicated folded proteins containing *α* helices, *β* strands, *β* turns, and unordered structures but lacked signals typical for triple helices. The recombinant *α*1 chains were further analyzed by negative staining electron microscopy. The purified wild-type *α*1 chains were seen as amorphous structures varying in size and shape, ranging from monomers to compact globular aggregates with a diameter of approx. 20 nm (Figure 4(e)). Similar structures were seen for the mutant *α*1 chain. However, here, the protein aggregates consistently had a larger diameter.

### 3.7. The Transfected Mutant *α*1 Chains Do Not Assemble to Collagen VI Tetramers in Collagen VI *α*1 Chain-Deficient WI-26 VA4 Cells and Instead Form Aggregates

**Figure 5:**
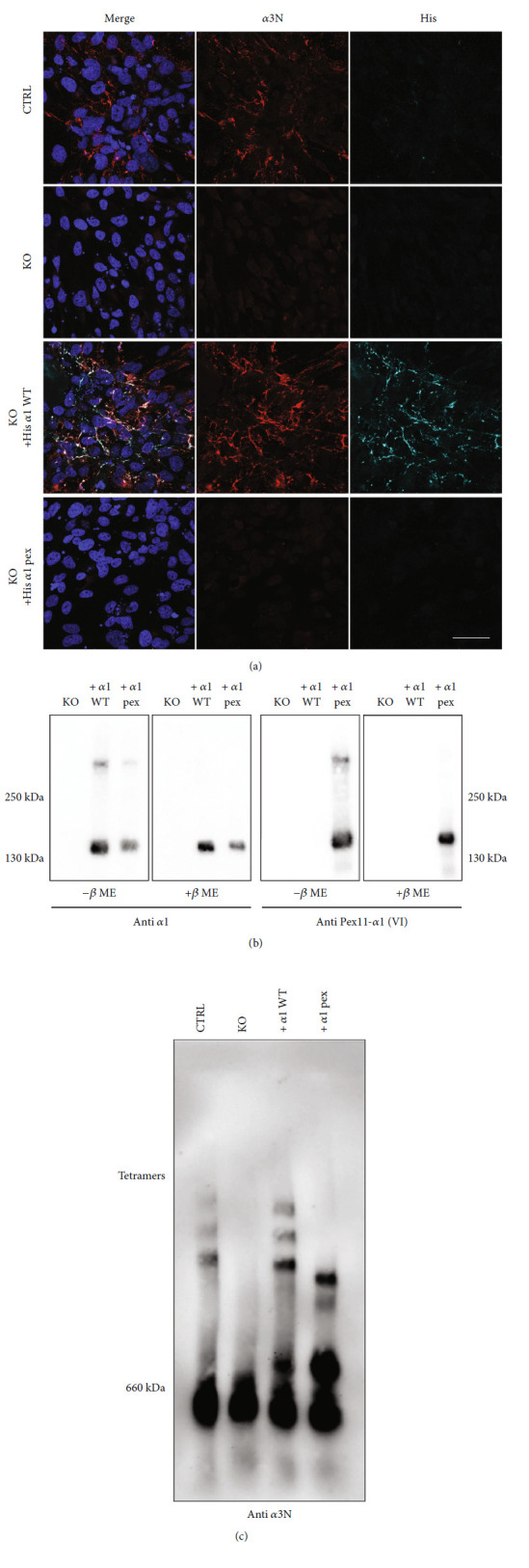
Recombinant mutant *α*1 chains do not assemble into collagen VI tetramers in WI-26 VA4 *COL6A1* KO cells. (a) Control WI26 cells (CTRL), *COL6A1* knock out (KO) and KO cells transfected with either the *α*1 wild-type (KO + His *α*1 WT) or mutant chain (KO + His *α*1 pex) were incubated for seven days in the presence of ascorbic acid. Labellings were performed with the *α*3N antibody to detect collagen VI microfibrils and the His antibody in order to localize the recombinant protein; scale bar = 50 *μ*m. (b) Supernatants from cells (KO and retransfected) were separated on a 6% SDS-PAGE gel under non-reducing (-*β*Me) or under reducing (+*β*Me) conditions and detected with antibodies against the *α*1 chains or the pseudoexon sequence (Pex). (c) Supernatants of the two cell lines were resolved on a composite agarose/polyacrylamide gel and tetramers were detected with the *α*3N antibody via immunoblot. The mutant chains cannot form tetramers but assemble into extracellular patches. These do not have the organized network structure seen for the wild-type chains.

**Figure 6:**
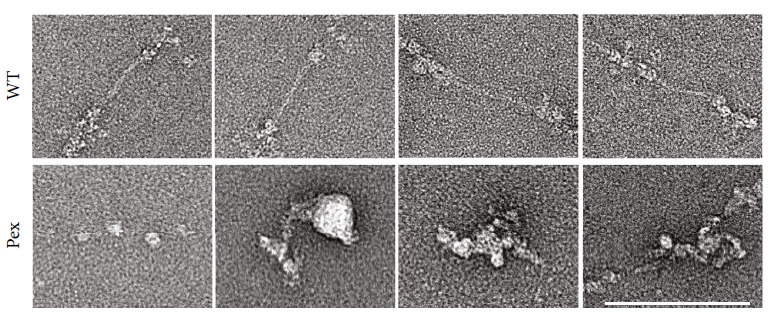
Negative staining electron micrographs of collagen VI purified from the supernatant of WI-26 VA4 *COL6A1* KO cells transfected with the *α*1 wild-type chain (WT) or the mutant chain (Pex) Scale bar = 100 nm. Rather than forming tetramers, the mutant chains produce unordered structures that range from globular aggregates to larger unordered aggregates.

As the mutant *α*1 chains could be recombinantly expressed, we generated a cell line expressing the mutant chains together with the wild-type *α*2 and *α*3 chains. This allowed us to study the consequences of the mutation under standardized conditions. We choose expression in human WI-26 VA4 cells, a lung fibroblast cell line that secretes collagen VI tetramers and forms a collagen VI-containing extracellular network (Figure 5(a); Supporting Information: Figure S1). We ablated the wild-type *COL6A1* alleles in the WI-26 VA4 cells by CRISPR-Cas (Supporting Information: Figure S1) and then transfected the cells with the mutant chain or with the wild-type *α*1 chain, serving as a control. Both chains were expressed (Figure 5(b)). Immunoblotting of composite agarose/polyacrylamide gels and immunofluorescence staining of *α*1 chain-deficient WI-26 VA4 cells transfected with the wild-type *α*1 chain clearly demonstrated that these cells secrete collagen VI tetramers (Figure 5(c)) and lay down a collagen VI network (Figure 5(a)). In contrast, the cells transfected with the mutant *α*1 chain do not assemble tetramers (Figure 5(c)). Instead, immunoblots with the *α*3N antibody detected a major band at approx. 660 kDa and an additional band between this position and the tetramer band (Figure 5(c)). In immunofluorescence microscopy of cells cultured on coverslips, extracellular patches stained for the mutant *α*1 chains were detected. However, an organized network, as formed by cells transfected with the wild-type *α*1 chain, was lacking (Figure 5(a)). Moreover, hardly any *α*3 chain signal was seen, confirming that collagen VI assembly is abolished when only the mutant version of the *α*1 chain is present. We also wanted to study the architecture of the aggregates formed by the secreted mutant *α*1 chains and to compare these with collagen VI assemblies formed by the wild-type *α*1 chain transfected WI-26 VA4 cells. For this purpose, affinity chromatography on a Strep-Tactin® XT column was employed, and the material containing recombinant *α*1 chains carrying the One-STrEP tag was subjected to electron microscopy after negative staining. The wild-type *α*1 chain-containing material showed the typical tetramers. In contrast, mutant *α*1 chain transfected cells produced unordered structures that varied in size and shape. They ranged from globular aggregates, similar to those derived from the mutant *α*1 chain transfected HEK293-EBNA cells, to larger irregular aggregates. As these larger aggregates were not produced in transfected HEK293-EBNA cells, they may contain other proteins in addition to the *α*1 chain (Figure 6).

## 4. Discussion

Collagen VI-related myopathies comprise a broad disease spectrum ranging from BM at the mild end to the severe UCMD. These myopathies are caused by mutations in the genes encoding the collagen VI *α*1, *α*2, or *α*3 chains. It was shown that the dysfunctional collagen VI has downstream effects on mitochondrial function [44] and autophagy and is associated with an increase of apoptosis in muscle [45]. The complex assembly of three different *α* chains to a network of collagen VI microfibrils involves multiple steps, both within the secretory pathway and outside the secreting cells. It is, therefore, difficult to predict with certainty by which mechanism a particular mutation finally causes a collagen VI myopathy. Only for some of the 453 pathogenic variations deposited in the Leiden open variation database [46] their impact on collagen VI assembly has been studied in detail. Interestingly, a recurrent *COL6A1* pseudoexon insertion was recently detected that is associated with an unusually progressive clinical course [34]. Here, we studied *in vitro* and *in vivo* how the mutant pseudoexon containing COL6A1 chains exert their dominant pathogenic effect.

The mutation lies in the N-terminal region of the triple helical domain [34], in the same region in which also other dominant mutations cluster [15]. Interestingly, not only point mutations of Gly in Gly-X-Y repeats but also inframe exon skipping mutations are located there. However, the pseudoexon insertion mutation we studied is unique, as it is the only known inframe intronic insertion in this region. In general, larger insertion mutations are rare in collagen VI myopathies. There is one example where a 16 amino acid residue insertion is present at the transition between the triple helix and the C1 domain of the *α*2 chain [47]. However, this dominant mutation causes mild BM, in contrast to the pseudoexon insertion mutation that causes severe UCMD. Just as the 24 amino acid insertion mutation, the Gly missense mutations in Gly-X-Y repeats [48] and the exon deletions in this region are dominant, [49, 50], which may point to a common pathomechanism. The wild-type mRNA is predominant in patient muscle, and only up to 27.5% of the mRNA contains the pseudoexon insertion, probably due to incomplete penetrance of mutant splice donor and cryptic splice acceptor [37]. Still, the severity of the disease is striking, indicating that the mutant chains have a strong dominant impact. Collagen VI normally assembles into tetramers consisting of *α*1, *α*2, and *α*3 chain-containing heterotrimeric triple helical monomers. Therefore, if the mutant chains are assembly competent, on average, at least one mutant chain must be present in each tetramer. This would be sufficient for the global expression of defective tetramers and could explain the phenotype. Such a pathomechanism has been proposed for the other dominant UCMD-causing mutations where the N-terminal region of the triple helix is affected [48–50]. However, we here clearly demonstrate that the mutant chains are not incorporated into collagen VI tetramers in any detectable amounts but are instead mainly independently secreted and deposited in the extracellular matrix. The secretion of mutant collagen VI *α*1 chains as nontriple helical polypeptides represents a novel finding. Interestingly, it seems that single, nonassembled collagen VI *α* chains can also be expressed and secreted by a number of human cancer cell lines and may be involved in cancer cell migration, invasion, and proliferation [51].

**Figure 7:**
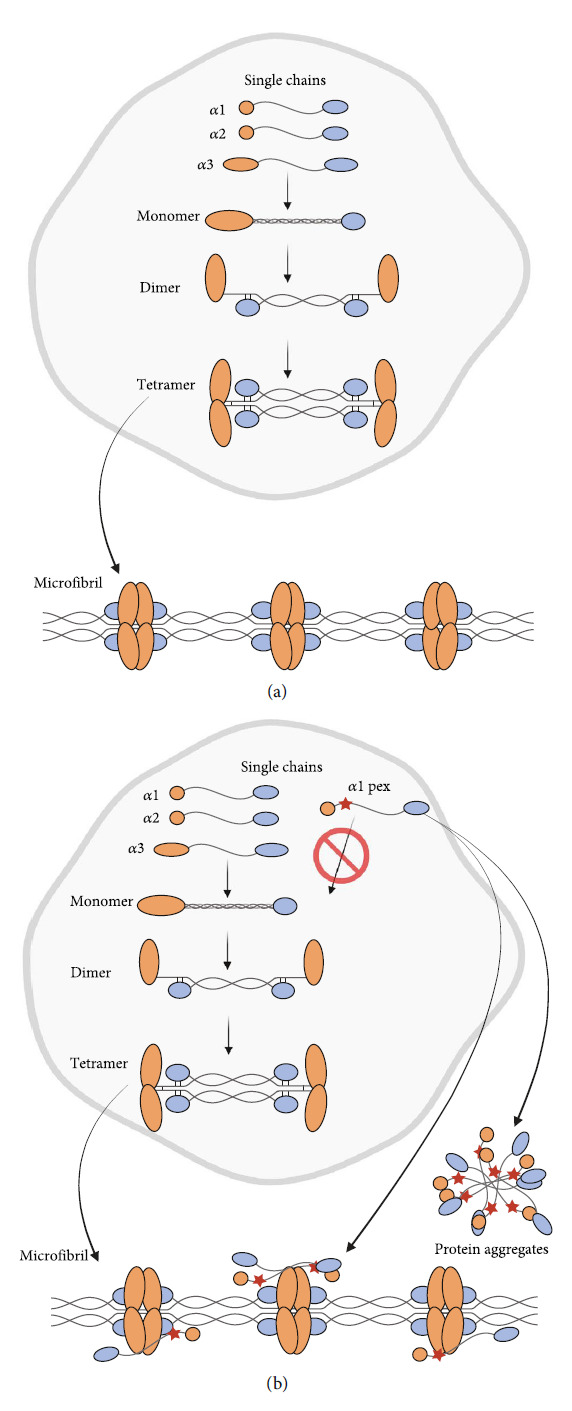
Schematic representation of the mutant effect. (a) Assembly of wild-type collagen VI microfibrils. Triple helical monomers (*α*1, *α*2, and *α*3) form dimers in an antiparallel manner that assemble laterally and form collagen VI tetramers. Tetramers are secreted and associated end-to-end by non-covalent interactions (for more detailed description see Introduction). (b) Mutant *α*1 chains (*α*1pex) carrying the pseudoexon insertion (red star) do not assemble with the other chains, are secreted, and form extracellular aggregates. In parallel, wild-type collagen VI microfibrils are formed that are decorated by mutant chains which may disturb their proper assembly and function. Schematic representation with changes from [15], created with http://BioRender.com.

Our studies indicate that the expression and secretion of single nontriple helical *α*1 chains is the basis for the deleterious effect. While the insertion of 24 amino acid residues into the triple-helix-domain prevents incorporation into properly folded collagen VI molecules, our data suggest that the deleterious effect is probably not caused by the inserted 24 amino acid sequence *per se*. Dominant mutations often lead to a gain of function or a gain of misfunction. Here, the gain of misfunction has two aspects: the expression of secreted, unordered, self-aggregating subunits and the disturbance of assembly of wild-type collagen VI microfibrils (Figure 7). Collagen VI is a VWA domain multimer, rather than a classical triple helical collagen molecule. VWA domains are protein-protein interaction modules that often tend to aggregate [52], when not in a physiological setting with their natural interaction partners. The aggregated mutant *α*1 chains are reminiscent of the protein deposits occurring in amyloid-forming diseases. Interestingly, in patients with Alzheimer's disease and in an Alzheimer's mouse model, the collagen VI *α*1 chain mRNA levels are elevated [53], and in a proteomic study, amyloid-positive human adipose tissue correlated with increased collagen VI [54]. In the case of amyloid light chain amyloidosis, collagens and glycosaminoglycans colocalized with protein aggregates and modulated amyloid formation [55]. As shown here by immunoelectron microscopy, mutant collagen VI *α*1 chains may disturb collagen VI microfibril assembly. This is likely to be due to their affinity for collagen VI microfibrils, perhaps mediated by the VWA domains. Moreover, the striking gradual increase of disease severity with time may be caused by an increasing aggregate deposition.

The generation of a pseudoexon-specific antibody was instrumental to our studies, as it enabled us to easily identify the mutant chains and distinguish them from the wild-type *α*1 chains. Such a tool is not available for other mutations that affect the assembly of collagen VI. Perhaps the secretion of assembly incompetent collagen VI *α* chains, caused by a mutation in the highly sensitive region of the collagen VI triple helix, may explain also other UCMD pathologies originating from this region. However, the corresponding secreted mutant collagen VI chains were not yet described, most likely as tools for their detection were not available. Therefore, it may be worthwhile to develop antibodies specific for these mutant chains and revisit the specific effect of the particular mutation. The fate of wild-type collagen VI chains, when another constituent chain is mutated or completely missing, is not well known. Although many studies used immunohistochemistry and immunoblots of composite agarose/polyacrylamide gels, the antibodies used may not have been sufficiently specific. The fate of each nonaffected chain should be monitored by chain-specific antibodies, even when normal tetramer formation occurs, and it should be determined in which form they are present. It is often difficult to obtain patient material to study the pathomechanism of a certain mutation. Here, we show that inactivation of a collagen VI *α* chain encoding gene in cultured WI-26 VA4 fibroblasts by CRISPR Cas and subsequent introduction of a construct expressing the mutant chain can be a valuable tool that may offer the possibility to study the efficacy of the treatment of an individual mutation *in vitro*.

Taken together, the expression and secretion of single VWA domain containing collagen VI *α*1 chains that tend to aggregate may have a strong impact on the pathogenesis of collagen VI-related myopathies. This may have been underestimated due to a lack of tools for detection. It will, therefore, be worthwhile to develop such tools and revisit the pathology of collagen VI myopathies with these.

## Data Availability

Data are in the manuscript and in the Supplementary Information files that are submitted alongside the manuscript.

## Supplementary Materials

Supplementary Materials Supplementary 1. Supplemental Figure 1: generation of *α*1 deficient WI-26 cell line. Supplementary 2. Supplemental Table 1: synthetic double-stranded DNA fragment.
