# Supplementary material for: The UCMD-Causing COL6A1 (c.930 + 189C > T) Intron Mutation Leads to the Secretion and Aggregation of Single Mutated Collagen VI α1 Chains
Source: Hum Mutat. 2023 Sep 6;2023:6892763. doi: 10.1155/2023/6892763 (PMC11919215; doi:10.1155/2023/6892763)
Supplement: Supplementary Materials — Supplementary 1. Supplemental Figure 1: generation of α1 deficient WI-26 cell line. Supplementary 2. Supplemental Table 1: synthetic double-stranded DNA fragment. [file 6892763.f1.pdf]

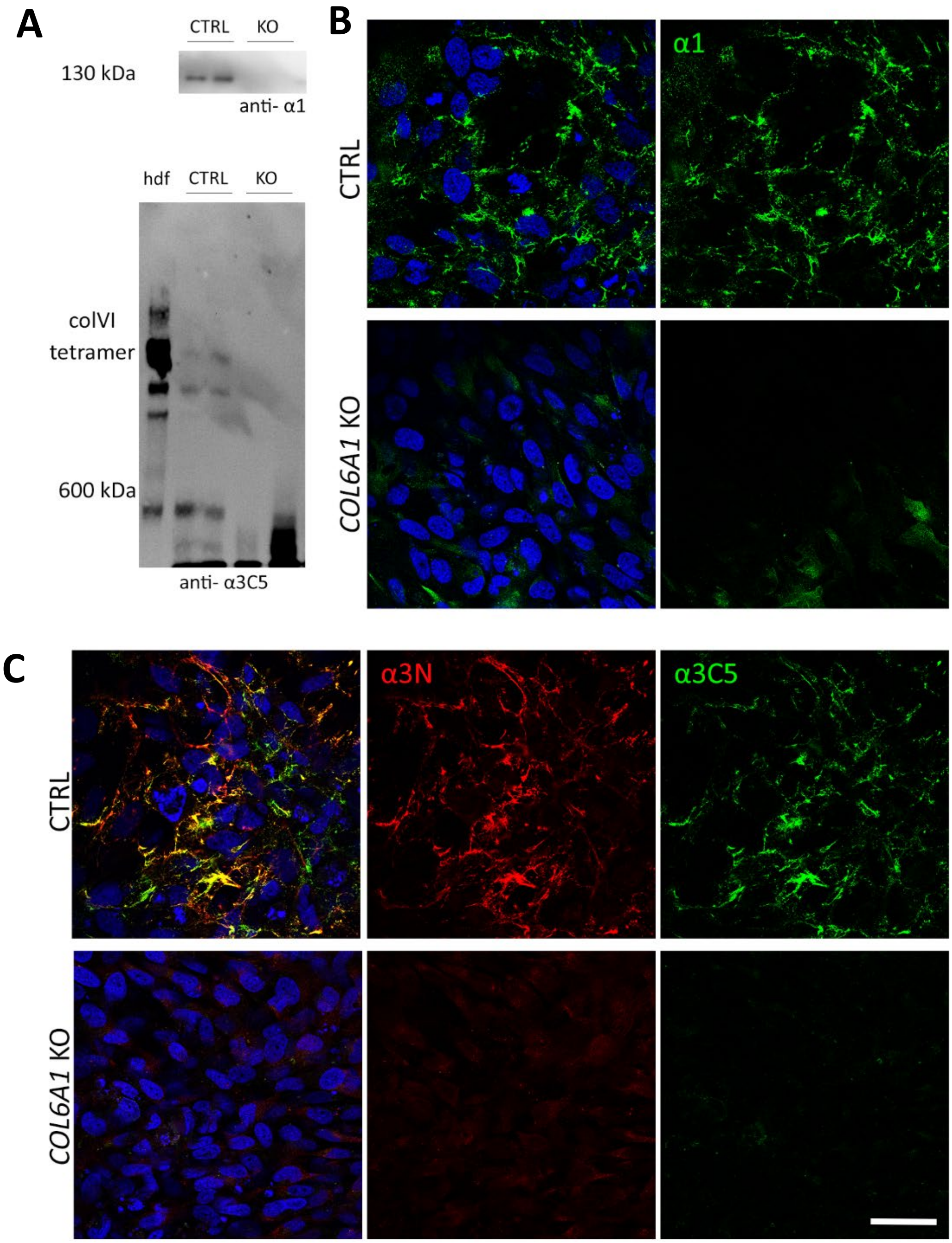

Figure S1: Generation of α1 deficient WI-26 VA4 cell line. (A) Supernatants from control WI-26 VA4 cells (CTRL) and COL6A1 knock out (KO) cells were separated on a 6 % SDS-PAGE gel (anti-α1) and on a composite agarose/polyacrylamide gel (anti-α3C5). hdf, supernatant from human dermal fibroblast cultures. (B,C) CTRL and KO cells were incubated for seven days in the presence of ascorbic acid. Labellings were performed with the α1 antibody (B) and the α3C5 and α3N antibody (C) to detect collagen VI microfibrils.

Table S1: Synthetic double stranded DNA fragment (Invitrogen GeneArt Strings).

Target organism	Gene	Protein	Sequence (5' – 3')	Restriction site(s)*
Human	*COL6A1*<br>*c.930+189C>T*	Col6α1pex	tataTTCGAATGCCAGCCTGCAAGAGGACCTCCGGGGCTCCGGGGC<br>GACCCCGGCTTTGAGGGAGAACGAGGCAAGCCGGGGCTCCCAGGA<br>GAGAAGGGAGAAGCCGGAGATCCTGGAAGACCCGGGGACCTCGG<br>ACCTGTTGGGTACCAGGGAATGAAGGGAGAAAAAGGGAGCCGTG<br>GGGAGAAGACCCGCTCCACCGCCCCTCGCCGTCCCCTCCATCTGGA<br>AGGACAAGGACAGCCACCCAGGCACCCAGCAAAGGGCTCCAGGGG<br>ACCCAAGGGCTACAAGGGAGAGAAGGGCAAGCGTGGCATCGACG<br>GGGTGGACGGCGTGAAGGGGGAGATGGGGTACCCAGGCCTGCCA<br>GGCTGCAAGGGCTCGCCCGGGTTTGACGGCATTCAAGGACCCCCTG<br>GCCCCAAGGGAGACCCCGGTGCCTTTGGACTGAAAGGAGAAAAGG<br>GCGAGCCTGGAGCTGACGGGGAGGCGGGGAGACCAGGGAGCTCG<br>GGACCATCTGGAGACGAGGGCCAGCCGGGAGAGCCTGGGCCCCCC<br>GGAGAGAAAGGAGAGGCGGGCGACGAGGGGAACCCAGGACCTGA<br>CGGTGCCCCCGGGGAGCGGGGTGGCCCTGGAGAGAGAGGACCAC<br>GGGGGACCCCAGGCACGCGGGGACCAAGAGGAGACCCTGGTGAA<br>GCTGGCCCGCAGGGTGATCAGGGAAGAGAAGGCCCCGTTGGTGTC<br>CCTGGAGACCCGGGCGAGGCTGGCCCTATCGGACCTAAAGGCTAC<br>CGAGGCGATGAGGGTCCCCCAGGGTCCGAGGGTGCCAGAGGAGC<br>CCCAGGACCTGCCGGACCCCCTGGAGACCCGGGGCTGATGGGTGA<br>AAGGGGAGAAGACGGCCCCGCTGGAAATGGCACCGAGGGCTTCCC<br>CGGCTTCCCCGGGTATCCGGGCAACAGGGGCGCTCCCGGGATAAA<br>CGGCACGAAGGGCTACCCCGGCCTCAAGGGGGACGAGGGAGAAG<br>CCGGGGACCCCGGAGACGATAACAACGACATTGCACCCCGAGGAG<br>TCAAAGGAGCAAAGGGGTACCGGGGTCCCGAGGGCCCCCAGGGA<br>CCCCCAGGACACCAAGGACCGCCTGGGCCGGACGAATGCGAGATT<br>TTGGACATCATCATGAAAATGTGCTCTTGCTGTGAATGCAAGTGCG<br>GCCCCATCGACCTCCTGTTCGTGCTGGACAGCTCAGAGAGCATTGG<br>CCTGCAGAACTTCGAGATTGCCAAGGACTTCGTCGTCAAGGTCATC<br>GACCGGCTGAGCCGGGACGAGCTGGTCAAGTTCGAGCCAGGGCAG<br>TCGTACGatat	BstBI<br>BsiWI

*restriction sites are *underlined*, the pseudoexon encoding sequence is given in *red.*
